# Hologram QSAR Studies of Antiprotozoal Activities of Sesquiterpene Lactones

**DOI:** 10.3390/molecules190710546

**Published:** 2014-07-18

**Authors:** Gustavo H. G. Trossini, Vinícius G. Maltarollo, Thomas J. Schmidt

**Affiliations:** 1 Faculdade de Ciências Farmacêuticas, Universidade de São Paulo, Av. Lineu Prestes, 580, 05508-000 São Paulo, Brazil; E-Mail: maltarollo@usp.br; 2 Institute of Pharmaceutical Biology and Phytochemistry (IPBP), University of Münster, Pharma Campus, Corrensstraße 48, D-48149 Münster, Germany; E-Mail: thomschm@uni-muenster.de

**Keywords:** HQSAR, sesquiterpene lactones, *Trypanosoma brucei*, *Trypanosoma cruzi*, *Leishmania donovani*, *Plasmodium falciparum*, antiprotozoal activity, fragment-based drug design

## Abstract

Infectious diseases such as trypanosomiasis and leishmaniasis are considered neglected tropical diseases due the lack for many years of research and development into new drug treatments besides the high incidence of mortality and the lack of current safe and effective drug therapies. Natural products such as sesquiterpene lactones have shown activity against *T. brucei* and *L. donovani*, the parasites responsible for these neglected diseases. To evaluate structure activity relationships, HQSAR models were constructed to relate a series of 40 sesquiterpene lactones (STLs) with activity against *T. brucei*, *T. cruzi*, *L. donovani* and *P. falciparum* and also with their cytotoxicity. All constructed models showed good internal (leave-one-out *q^2^* values ranging from 0.637 to 0.775) and external validation coefficients (*r^2^_test_* values ranging from 0.653 to 0.944). From HQSAR contribution maps, several differences between the most and least potent compounds were found. The fragment contribution of PLS-generated models confirmed the results of previous QSAR studies that the presence of α,β-unsatured carbonyl groups is fundamental to biological activity. QSAR models for the activity of these compounds against *T. cruzi*, *L. donovani* and *P. falciparum* are reported here for the first time. The constructed HQSAR models are suitable to predict the activity of untested STLs.

## 1. Introduction

Nowadays, several diseases caused by protozoan parasites such as leishamaniases, trypanosomiases (Chagas Disease and African Sleeping sickness) and malaria represent major health risks in developing countries. Leishmaniases and trypanosomiases have few available drug therapies and the development of anti-malarial compounds is also urgently needed due to rapidly emerging resistance of the parasites against existing drugs. Is estimated that the infections by *Trypanosoma* and *Leishmania* are responsible for over a million deaths per year. Their treatment by drugs is complicated by severe side effects due to the high toxicity of available drugs. Due to the lack of research and development of new drugs over many decades, these diseases are considered “neglected tropical diseases” [1,2,3,4,5]. There is thus an urgent need for development of new therapeuticals against these diseases. Many classes of chemicals have been tested against these parasites. Among them, natural products and, particularly, sesquiterpene lactones (STLs) have shown interesting activities [6,7].

In a previous study [8], *in vitro* activity data for 40 sesquiterpene lactones (STLs) against *Trypanosoma brucei rhodesiense* (the etiologic agent of East African sleeping sickness; Tbr), *T. cruzi* (Chagas Disease; Tcr), *Leishmania donovani* (visceral leishmaniasis, Kala-Azar; Ldon) as well as *Plasmodium falciparum* (tropical malaria; Pfc) were reported. Quantitative structure-activity relationship (QSAR) models for the activity against *T. brucei rhodesiense* and for the cytotoxic activity of these compounds against L6 rat skeletal myoblast cells were presented. It was found that the biological effects against the protozoan parasites were all correlated significantly with cytotoxicity against the mammalian control cells. It was not possible at that time and with the methods used for QSAR modelling to clearly define a structural basis for selectivity against the parasites [8]. QSAR approaches are considered powerful tools in lead identification as well as optimization [9] in cases where the bioactivity of congeneric sets of compounds is known. Even though QSAR methods have been applied to STLs successfully for several bioactivities [10,11,12,13] it remained a challenge to construct validated models of anti-protozoal activity of STLs against *T. cruzi*, *L. donovani* and *P. falciparum* [8,14].

The main objective of our present work is therefore to apply the hologram quantitative structure-activity relationship (HQSAR) approach to construct comparable models for all four mentioned protozoa and cytotoxicity and to employ the molecular fragment information of the generated models to analyze the structural basis for the antiprotozoal activity and cytotoxicity of the compounds in this data set in order to find possible reasons for the selectivity observed with some of the STLs.

## 2. Results and Discussion

**Figure 1:**
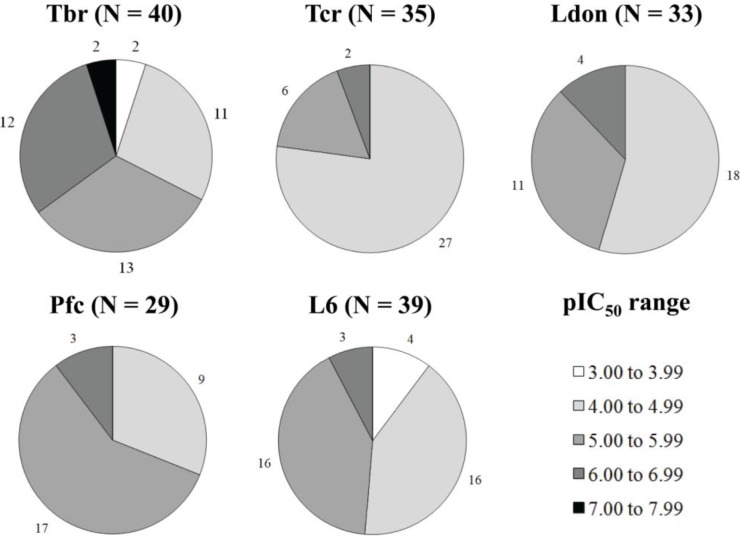
pIC_50_ distribution of the dataset of 40 STLs over the five biological activities under study. Each graph represents the respective number of compounds with measured pIC_50_ (N) values in a particular concentration range against each tested parasite and cytotoxicity (L6).

As biological activities against *T.brucei* and L6 cover a range of at least three logarithmic units, as shown by Figure 1, and all data within each activity set were determined under identical experimental conditions [8], the dataset is deemed suitable for QSAR studies. The biological activities against *T. cruzi*, *L. donovani* and *P. falciparum* only cover 2.25, 1.90 and 1.92 logarithmic units, respectively. This is not the ideal scenario to construct HQSAR models, but we decided to construct these three models in order to support the results generated by Tbr and L6 models.

Initially, the HQSAR models with 16 series of fragment distinction and fixed fragment size (4 to 7 atoms) were generated for each series of biological activity (*T. brucei rhodesiense*, *T. cruzi*, *L. donovani*, *P. falciparum* and L6 cytotoxicity). The five best models for each dependent variable are presented in Table 1.

The initial search for the fragment distinction that best represents each biological activity shows that the model employing fragments based on atoms, bonds and connections (A/B/C) provides the best description for anti-*T. brucei* activity (*q^2^* = 0.637). The best models for *T. cruzi* and *P. falciparum* were obtained employing fragments based on atoms and connections (A/C) with cross-validated correlation coefficients (*q^2^*) equal to 0.721 and 0.703, respectively. Finally, best HQSAR models for *L. donovani* (*q^2^* = 0.775) and cytotoxicity (*q^2^* = 0.647) employed fragment distinction based on atoms, connections, chirality and H-bond donor/acceptor groups (A/C/Ch/DA). In general, these initial results indicate that both anti-Ldon activity and cytotoxicity could be influenced more strongly by H-bond interactions and stereoselectivity since the best Ldon and L6 models were the only ones constructed with Ch and DA flags in fragment distinction.

**Table 1:** 5 Best HQSAR models with fragment size equal to 4 to 7 atoms.

*Tbr HQSAR models*						
F_dist_	*q^2^*	SEV	*r^2^*	SEE	HL	PC
**A/B/C**	**0.637**	**0.576**	**0.822**	**0.404**	**53**	**4**
A/C/Ch	0.619	0.591	0.823	0.403	83	4
A/C/Ch/DA	0.601	0.604	0.871	0.343	53	4
A/C	0.577	0.622	0.835	0.389	71	4
A/B/C/Ch	0.573	0.625	0.833	0.391	307	4
***Tcr HQSAR models***						
**F_dist_**	***q^2^***	**SEV**	***r^2^***	**SEE**	**HL**	**PC**
**A/C**	**0.721**	**0.297**	**0.939**	**0.139**	**71**	**6**
A/B/C	0.697	0.309	0.950	0.125	53	6
A/B	0.695	0.303	0.884	0.187	353	5
A/B/C/DA	0.694	0.304	0.922	0.153	71	5
A/C/Ch	0.667	0.317	0.919	0.156	151	5
***Ldon HQSAR models***						
**F_dist_**	***q^2^***	**SEV**	***r^2^***	**SEE**	**HL**	**PC**
**A/C/Ch/DA**	**0.775**	**0.279**	**0.972**	**0.098**	**83**	**6**
A/C/DA	0.768	0.283	0.959	0.119	61	6
A/B	0.731	0.289	0.920	0.158	71	4
A/C	0.727	0.292	0.892	0.183	61	4
A/C/Ch	0.707	0.319	0.970	0.103	199	6
***Pfc HQSAR models***						
**F_dist_**	***q^2^***	**SEV**	***r^2^***	**SEE**	**HL**	**PC**
**A/C**	**0.703**	**0.254**	**0.950**	**0.104**	**83**	**6**
A/B/C	0.684	0.262	0.951	0.104	307	6
A/C/Ch	0.682	0.263	0.948	0.106	61	6
A/C/DA	0.676	0.265	0.954	0.100	151	6
A/B/C/DA	0.668	0.253	0.888	0.147	151	4
***L6 HQSAR models***						
**F_dist_**	***q^2^***	**SEV**	***r^2^***	**SEE**	**HL**	**PC**
**A/C/Ch/DA**	**0.647**	**0.343**	**0.893**	**0.189**	**61**	**5**
A/B/C/Ch	0.646	0.351	0.907	0.180	61	6
A/C	0.636	0.348	0.852	0.222	257	5
A/B/C/H	0.633	0.357	0.912	0.175	353	6
A/B/C	0.629	0.351	0.862	0.215	353	5

F_dist_: fragment distinction; HL: hologram length; PC: number of PLS principal components; standard error of validation; SEE: standard error of estimation.

After this step, the fragment distinction of the best models was fixed and then a variation of fragment size was employed in order to analyze the influence of this parameter on statistical results. For each model (Tbr, Tcr, Ldon, Pfc and L6 models), we tested the fragment sizes with: 1 to 4 atoms, 2 to 5 atoms, 3 to 6 atoms, 4 to 7 atoms (tested in first step), 5 to 8 atoms, 6 to 9 atoms, 7 to 10 atoms and 8 to 11 atoms. All results of this second step are shown in Table 2.

**Table 2:** HQSAR models with fragment size variations from 1–4 atoms to 8–11 atoms.

*Tbr HQSAR models with F_dist_ = A/B/C*						
F_size_ (atoms)	*q^2^*	SEV	*r^2^*	SEE	HL	PC
1 to 4	0.407	0.736	0.708	0.517	97	4
2 to 5	0.547	0.644	0.761	0.467	83	4
3 to 6	0.546	0.644	0.808	0.419	97	4
**4 to 7**	**0.637**	**0.576**	**0.822**	**0.404**	**53**	**4**
5 to 8	0.588	0.614	0.833	0.391	307	4
6 to 9	0.565	0.631	0.817	0.409	53	4
7 to 10	0.519	0.663	0.826	0.398	61	4
8 to 11	0.480	0.690	0.819	0.406	151	4
***Tcr HQSAR models with F_dist_ = A/C***						
**F_size_ (atoms)**	***q^2^***	**SEV**	***r^2^***	**SEE**	**HL**	**PC**
1 to 4	0.330	0.440	0.640	0.322	53	4
2 to 5	0.518	0.381	0.825	0.230	53	5
4 to 7	0.721	0.297	0.939	0.139	71	6
3 to 6	0.637	0.339	0.934	0.145	199	6
5 to 8	0.741	0.280	0.948	0.126	151	5
6 to 9	0.729	0.286	0.953	0.120	151	5
**7 to 10**	**0.748**	**0.282**	**0.965**	**0.106**	**151**	**6**
8 to 11	0.689	0.299	0.923	0.149	353	4
***Ldon HQSAR models with F_dist_ = A/C/Ch/DA***						
**F_size_ (atoms)**	***q^2^***	**SEV**	***r^2^***	**SEE**	**HL**	**PC**
1 to 4	0.542	0.398	0.881	0.203	151	6
2 to 5	0.618	0.364	0.904	0.182	83	6
3 to 6	0.706	0.319	0.966	0.108	151	6
**4 to 7**	**0.775**	**0.279**	**0.972**	**0.098**	**83**	**6**
5 to 8	0.770	0.282	0.982	0.080	199	6
6 to 9	0.747	0.296	0.976	0.091	199	6
7 to 10	0.733	0.296	0.968	0.103	307	5
8 to 11	0.716	0.305	0.965	0.106	257	5
***Pfc HQSAR models F_dist_ = A/C***						
**F_size_ (atoms)**	***q^2^***	**SEV**	***r^2^***	**SEE**	**HL**	**PC**
1 to 4	0.458	0.333	0.809	0.197	59	5
2 to 5	0.615	0.280	0.875	0.160	61	5
3 to 6	0.730	0.242	0.944	0.110	71	6
4 to 7	0.703	0.254	0.950	0.104	83	6
5 to 8	0.706	0.253	0.961	0.092	307	6
6 to 9	0.683	0.262	0.965	0.087	353	6
**7 to 10**	**0.736**	**0.232**	**0.960**	**0.090**	**97**	**5**
8 to 11	0.732	0.241	0.982	0.063	353	6
***L6 HQSAR models F_dist_* = A/C/Ch/DA**						
**F_size_ (atoms)**	***q^2^***	**SEV**	***r^2^***	**SEE**	**HL**	**PC**
1 to 4	0.204	0.504	0.713	0.303	61	4
2 to 5	0.465	0.422	0.856	0.219	71	5
3 to 6	0.568	0.371	0.843	0.224	307	4
4 to 7	0.647	0.343	0.893	0.189	61	5
**5 to 8**	**0.673**	**0.337**	**0.952**	**0.129**	**71**	**6**
6 to 9	0.619	0.363	0.966	0.109	59	6
7 to 10	0.549	0.379	0.889	0.188	151	4
8 to 11	0.583	0.373	0.948	0.131	151	5

F_dist_: fragment distinction; F_size_: fragment distinction; HL: hologram length; PC: number of PLS principal components; standard error of validation; SEE: standard error of estimation.

After the analysis of the influence of fragment distinction and size, hologram length and number of PCs on the statistical parameters, we evaluated the quality of the constructed models by internal and external validations.

**Figure 2:**
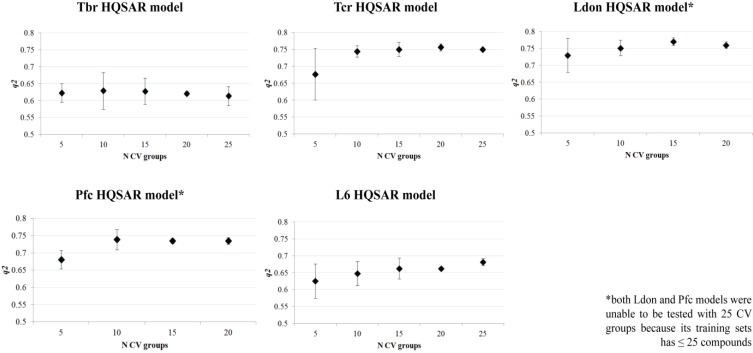
Robustness test of the five constructed HQSAR models.

**Figure 3:**
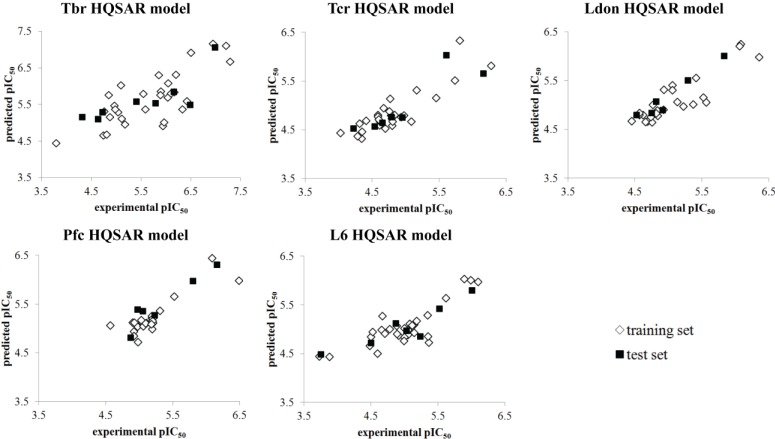
Experimental *versus* predicted pIC_50_ values of training and test sets of all constructed HQSAR models.

The robustness test (Figure 2) suggests that all constructed models have acceptable internal consistency since all average *q^2^* values for each number of cross-validation groups were higher than 0.6. In order to certify that all models are completely validated, the *r^2^_test_* value was calculated for each model and the residues of prediction were also considered in external validation. Table 3 summarizes all parameters of the constructed HQSAR models as well all statistical results of internal and external validations. Figure 3 displays the experimental *versus* predicted pIC_50_ values of all HQSAR models.

It is important to note that compound 17 in the *T. brucei* model, compounds 19 and 34 in the *L. donovani* model, and compounds 24 and 26 in the cytotoxicity model are considered outliers due to their high values of both CV and external validation residuals (residuals > 1.50 log units)

**Table 3:** Comparison of statistical results of all five constructed HQSAR models.

	HQSAR Models				
	Tbr	Tcr	Ldon	Pfc	L6
F_dist_	A/B/C	A/C	A/C/Ch/DA	A/C	A/C/Ch/DA
F_size_	4–7 atoms	7–10 atoms	4–7 atoms	7–10 atoms	5–8 atoms
HL	53	151	83	97	71
PC	4	6	5	5	6
N	31	28	25	23	30
*q^2^_LOO_*	0.637	0.748	0.775	0.736	0.673
SEV	0.576	0.282	0.279	0.232	0.337
*q^2^_CV_*	0.623 ± 0.03	0.736 ± 0.03	0.753 ± 0.02	0.722 ± 0.02	0.656 ± 0.03
*r^2^*	0.822	0.965	0.972	0.960	0.952
SEE	0.404	0.106	0.098	0.090	0.129
*r^2^_test_*	0.653	0.790	0.944	0.897	0.831

F_dist_: fragment distinction; F_size_: fragment size; HL: hologram lenght; PC: number of PLS principal components; N: number of compounds of training set; SEV: standard error of validation; SEE: standard error of estimation.

These compounds were removed from the respective data sets and the modelling repeated without them, in order to avoid distortions in the models. Manifold reasons may lead to the behavior of particular compounds as outliers [15] on which to speculate here for each case in detail does not appear useful. From the results of external validations, we can note that all constructed models have acceptable values of external validation correlation coefficients and residuals of prediction for all test set compounds lower than 1 logarithmic unit (Supplementary Table S6). All generated models including fragment distinction search and fragment size evaluation for the five sets of biological data are available in Supplementary Tables S1–S5.

Therefore, both the LOO and CV internal validation methods as well as the external validation provide results which indicate that all constructed HQSAR models and their respective fragments information are suitable to explain the anti-protozoal and cytotoxic activities.

**Figure 4:**
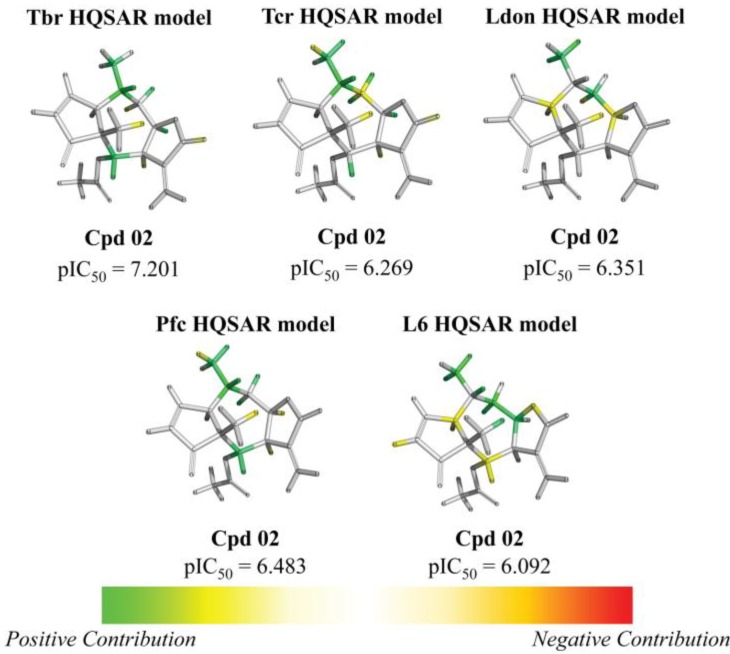
HQSAR maps of positive contribution for all 5 constructed HQSAR models.

From the contribution maps of compound **2**, one of the most potent compounds in each HQSAR model (Figure 4), it becomes clear that the 7-membered ring with one of the attached methyl groups is assigned a positive contribution to biological activity by each of the HQSAR models. Quite notably, the oxygen atom in the butyrolactone ring only shows a positive contribution to the cytotoxicity model, indicating that this atom (or the butyrolactone moiety) could be related to an important difference between anti-protozoal and toxic activities of the compounds in this data set. The lactone carbonyl oxygen atom contributes positively to Tbr and Tcr models.

**Figure 5:**
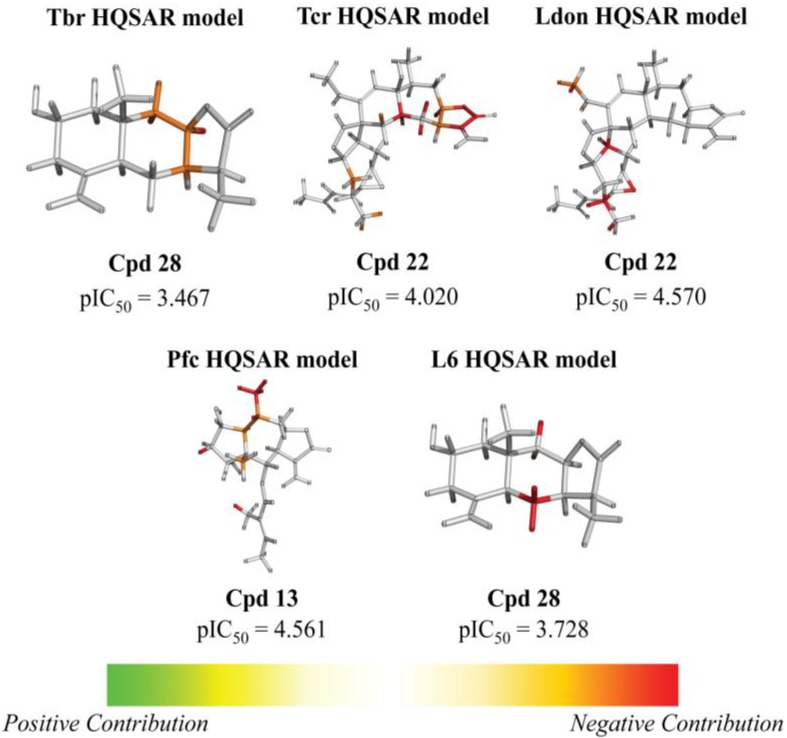
HQSAR maps of negative contribution for all five constructed HQSAR models.

Analyzing the contribution maps of the five constructed models (Figure 5), the 6-membered ring contributes negatively to *T. brucei*, *P. falciparum* and cytotoxic activities. The butyrolactone moiety (except the oxygen atom of carbonyl group) contributes negatively to anti *T. cruzi* activity. The oxygen atom of oxirane group contributes negatively to the *L. donovani* HQSAR model.

On the background of previous QSAR analyses of this data set, it could be expected that all HQSAR models should be influenced by similar parameters and lead to similar contribution maps since the pairwise correlation between the sets of biological activity values is quite high (higher than 69%, Supplementary Table S7) [8]. However, this is not the case so that the information provided by the contribution maps of the individual models could be useful to identify differences, especially between cytotoxicity and the anti-protozoal activities. Even though the differences between the models for the antiparasitic and cytotoxic activities may be subtle and difficult to interpret in detail due to the complexity of the applied descriptors, it is noteworthy that these differences exist and thus represent a possibility to rationalize the structural reasons for the selectivity of some compounds against the parasites.

**Figure 6:**
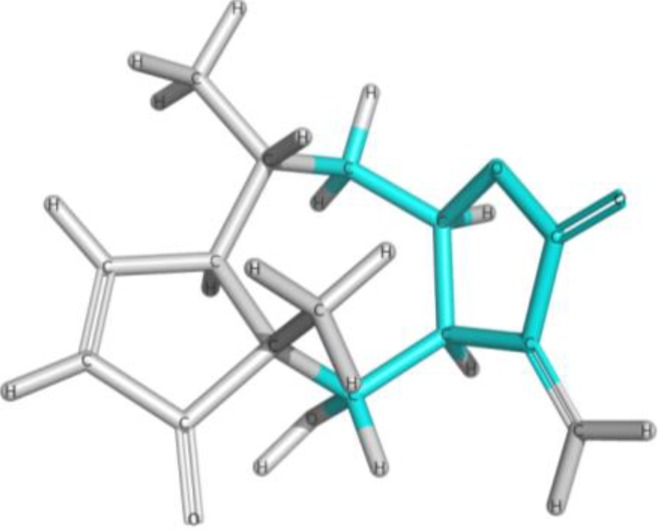
Maximum common structure (cyan atoms of compound 01) of dataset calculated by all HQSAR models.

We calculated the maximum common structure (MCS) with the HQSAR module (Figure 6, MCS colored in cyan). This MCS comprises the butyrolactone moiety along with two carbon atoms of the attached ring system. The α-methylene group, although present in most compounds, is not part of the MCS since compounds 5, 6, 7 and 35 are 11,13-dihydro derivatives, *i.e.*, they have a methyl group instead of the =CH_2_ group. Apart from this, compound 23 has a cyclic substituent at this position. Compounds 5, 6 and 7 are pseudoguaianolides bearing another α,β-unsatured carbonyl system, *i.e.*, a cyclopentenone moiety located on the opposite side of the molecule. Compounds 23 and 35 do not contain any α,β-unsatured carbonyl system and both show very low activity against Tbr and also no significant cytotoxicity (pIC_50_ values equal to 3.79 and 4.31, respectively). Therefore, our HQSAR studies indicate that the presence of α,β-unsatured carbonyl system could be considered a common scaffold which is generally related to biological activity, while the fragments with positive and negative contributions (Figure 4 and Figure 5) could be related to the differences of pIC_50_ in each model.

In order to perform an analysis of the anti-Tbr HQSAR model in terms of statistical influence of particular fragments on biological activity, we extracted the information about the fragments with highest positive and negative contributions to biological activity from this model (Table 4).

**Table 4:** List of fragments with highest positive and negative contribution to Tbr HQSAR model; X atoms are the connectivity flag and are not considered part of fragment.

**Frag 01**	**Frag 02**	**Frag 03**	Frag 04 *	Frag 05 *
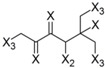	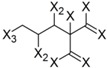	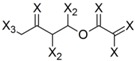	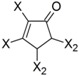	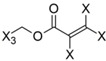
0.025	0.025	0.025	0.022	0.022
**Frag 06**	**Frag 07**	**Frag 08**	Frag 09 *	Frag 10 *
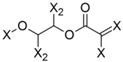	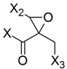	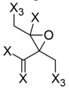	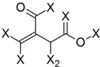	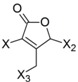
−0.016	−0.016	−0.016	−0.016	−0.016

* Fragments containing explicit α,β-unsatured carbonyl system with highest contribution values.

From the results obtained, it is possible to note that two of three fragments with highest contribution to biological activity (fragments 01 and 03) have two sp^2^ carbon atoms bonded directly to each other which would be characteristic of an α,β-unsatured carbonyl system. Fragment 04 is the fragment with an explicit α,β-unsatured carbonyl system with highest positive contribution to the model and this fragment is exactly the substructure present in compounds 01–08 which have the highest anti-*T. brucei* activities. Fragment 05 is one example of a fragment encoding the butyrolactone moiety indicating that this group also contributes positively to biological activity. There are also fragments containing an α,β-unsatured carbonyl system that show a negative contribution to the model (fragments 09, 10) but, in general terms, the values of their contribution to biological activity are lower than the positive ones, indicating that positive contributions have a higher statistical significance to this HQSAR model. From fragments with negative contributions (fragments 07, 08), it is possible to note that an epoxide group contributes negatively to anti-Tbr activity. As previously described, α,β-unsatured carbonyl systems such as the α-methylene-γ-lactone and cyclopentenone moiety are of major influence on biological activity of STLs, not only with respect to their antiprotozoal and cytotoxic activity. [8,10,11,14,16,17,18].

In comparison to recent descriptor-based QSARs models for *T. brucei* activity and cytotoxicity constructed by Schmidt *et al.* [7], the obtained results in HQSAR suggest similar physicochemical interpretations. The positive contribution of methylcycloheptane (as part of a pseudoguaianolide skeleton) to all models suggests a positive influence of this ring system on activity that may be due to steric or hydrophobic factors since the cyclohexane system as present in the eudesmanolides showed a negative contribution to biological activity for both Tbr and L6 models.

The two fragments with the highest contribution to the Tbr model represent alkene structures which are also hydrophobic groups. These results corroborate the positive contribution of hydrophobicity to anti-Tbr activity.

In summary, our HQSAR models showed once more that α,β-unsatured groups are fundamental to biological activity of STLs, in accordance with several previous works [7,8,9,10,11,12,13]. Furthermore, the methyl-cycloheptane ring as well as further hydrophobic groups appear to be responsible for higher levels of biological activity, indicating that the potency of the studied compounds could be related to cellular permeation mechanisms.

**Figure 7:**
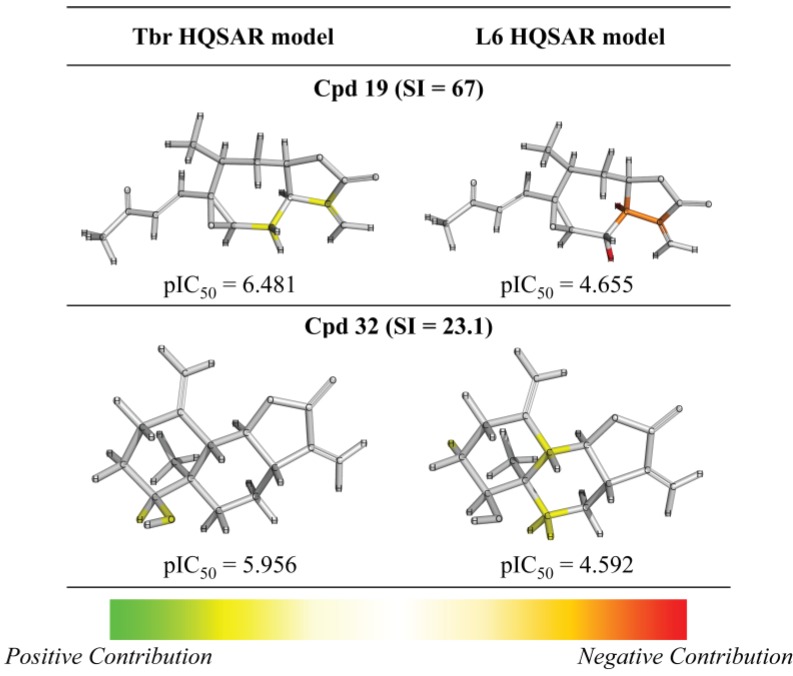
HQSAR maps of most selective compounds.

After the analyses of HQSAR maps and the influence of fragments for most and less potent compounds, we also analyzed the HQSAR maps of the compounds with highest selectivity indices (SI) for *T. brucei* (compounds 19, 24 and 32) and lowest SI (compounds 26, 25 and 28). We generated these maps with Tbr and L6 models in order to verify the influence of fragments for both biological activities as a strategy to study the selectivity. From the maps of compounds 24, 25, 26 and 28 we cannot verify significant differences that could explain the selectivity of the lack of it (Supplementary Figure S1). The maps of compounds 19 and 32 are shown by Figure 7.

From Figure 7, we can note: (i) the contribution maps of compound 19, the most *T. brucei* selective, indicates that de C-atoms of the α,β-unsatured carbonyl system and the 7-membered ring contribute positively to the Tbr model but negatively to toxicity. Therefore, this compound could be considered a lead for the development of new chemical entities with antiprotozoal activity and low toxicity; (ii) the contribution map for compound 32 indicates that the 6-membered ring contributes positively to toxicity. From this information, it is possible to note that this fragment is present in compounds with lower antiprotozoal activity and also could lead to increased toxicity; (iii) the O atom of the hydroxy group of the distal ring of compound 32 contributes positively to anti-*T. brucei* activity, indicating that compounds with an –OH group at this position could be tested due the low influence of this fragment on toxicity.

## 3. Experimental Section

### 3.1. Data Set

The data set used for the HQSAR studies contains 40 sesquiterpene lactones with their antiprotozoal activity against *Trypanosoma brucei rhodesiense* (Tbr), *Trypanosoma cruzi* (Tcr), *Leishmania donovani* (Ldon) and *Plasmodium falciparum* (Pfc), as well as cytotoxicity against L6 rat skeletal myoblasts (Table 5) [8]. The biological activity data were reported as micromolar IC_50_ values which were converted to molar pIC_50_ (−logIC_50_) and used as dependent variables in the QSAR model development (Table 5). The chemical structures were drawn in the 2D format and converted to 3D, using the Sybyl X 2.0 package [19]. The studied compounds were divided into training and test sets containing 80% and 20%, respectively, of the total number of compounds of each dataset (a set with certain compounds with specific biological activity measurement) in order to construct the HQSAR models and to perform external validations. The dataset split step was performed in such a manner that the entire range of pIC_50_ values was covered by test set compounds, also taking into account the structural homogeneity of training and test sets. Thus, both training and test set compounds were inside the two dimensional Y (biological activity) and X (fragment) spaces.

**Table 5:** Structures of dataset compounds and their pIC_50_ values. Selectivity indices (SI) are defined as SI = IC_50_(L6)/IC_50_(parasite) and showed between parenthesis.

*Pseudoguaianolides*																				
Cpd	Structure				R					Tbr				Tcr			Ldon		Pfc	L6
**1**	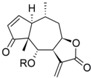				H					7.284 (19.1)				6.158 (1.4)			n.a.		n.a.	6.003
**2**					ac					7.201 (12.9)				6.269 (1.5)			6.351 (1.8)		6.483 (2.5)	6.092
**3**					i-butyryl					6.979 (9.8)				5.805 (0.7)			6.077 (1.2)		6.155 (1.5)	5.987
**4**					i-valeryl					6.936 (11.2)				5.606 (0.5)			6.060 (1.5)		6.085 (1.6)	5.887
**5**	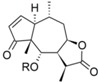				H					6.164 (13.0)				4.668 (0.4)			5.415 (2.3)		5.516 (2.9)	5.051
**6**					Ac					5.849 (2.2)				5.159 (0.4)			n.a.		n.a.	5.515
**7**					i-valeryl					6.040 (5.0)				5.452 (1.3)			5.831 (3.1)		5.795 (2.9)	5.339
**8**	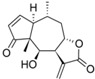				-					6.496 (7.7)				5.728 (1.3)			n.a		n.a.	5.612
**9**	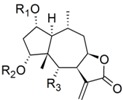	Ac	H				OH			5.033 (1.3)				4.339 (0.3)			n.a.		n.a.	4.911
**10**		Ac	H				H			5.174 (0.7)				4.690 (0.2)			4.912 (0.4)		5.190 (0.7)	5.357
**11**		H	H				H			4.736 (1.7)				4.278 (0.6)			4.686 (1.5)		4.916 (2.6)	4.496
**12**		H	tig				OH			4.961 (1.5)				4.308 (0.3)			4.746 (0.9)		4.975 (1.6)	4.778
**13**		H	H				Otig			4.716 (1.7)				4.221 (0.5)			4.838 (2.2)		4.561 (1.2)	4.498
**14**		Ac	ac				H			5.930 (7.2)				4.834 (0.6)			5.371 (2.0)		5.049 (0.9)	5.074
**15**		Ac	H				Oac			5.402 (1.8)				4.788 (0.4)			5.062 (0.8)		5.223 (1.2)	5.138
**16**	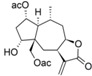	-	-				-			4.866 (1.6)				4.348 (0.5)			4.927 (1.8)		4.972 (2.0)	4.666
***Xanthanolides***																				
**Cpd**	**Structure**									**Tbr**				**Tcr**			**Ldon**		**Pfc**	**L6**
**17**	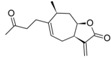									4.207 (2.9)				n.a.			4.623 (7.5)		n.a.	3.748
**18**	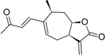									5.535 (10.4)				4.581 (1.2)			4.753 (1.7)		5.107 (3.9)	4.520
**19**	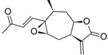									6.481 (67.0)				4.948 (2.0)			6.223 (37.0)		5.186 (3.4)	4.655
**20**	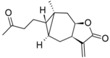									4.799 (1.3)				4.409 (0.5)			4.836 (1.4)		4.896 (1.6)	4.702
***Modified Xanthanolides***																				
**Cpd**	**Structure**									**Tbr**				**Tcr**			**Ldon**		**Pfc**	**L6**
**21**	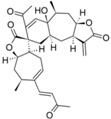									6.195 (14.8)				4.794 (0.6)			4.657 (0.4)		5.304 (1.9)	5.026
**22**	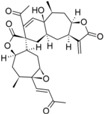									5.890 (10.3)				4.020 (0.1)			4.570 (0.5)		5.185 (2.0)	4.877
**23**	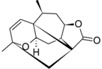									3.790				n.a.			4.812		n.a.	n.a.
**24**	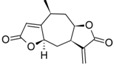									5.086 (52.1)				n.a.			4.568 (15.8)		n.a.	3.369
***Eudesmanolides***																				
**Cpd**	**Structure**					**R**				**Tbr**				**Tcr**			**Ldon**		**Pfc**	**L6**
**25**	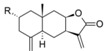					H				4.627 (0.2)				4.652 (0.2)			n.a.		n.a.	5.348
**26**						OH				5.108 (0.1)				4.606 (0.0)			n.a.		n.a.	6.023
**27**						Oac				4.970 (0.8)				4.614 (0.4)			4.930 (0.8)		5.036 (1.0)	5.050
**28**	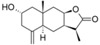					-				3.467 (0.5)				n.a.			n.a.		n.a.	3.728
**29**	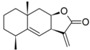					-				5.581 (2.3)				5.081 (0.7)			5.519 (2.0)		5.194 (0.9)	5.223
**30**	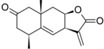					-				5.795 (6.4)				4.763 (0.6)			5.061 (1.2)		4.971 (1.0)	4.992
**31**	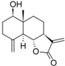					-				4.749 (1.9)				4.539 (1.1)			5.221 (5.5)		4.919 (2.8)	4.479
**32**	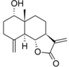					-				5.956 (23.1)				4.582 (1.0)			5.134 (3.5)		4.866 (1.9)	4.592
***Germacranolides***																				
**Cpd**	**Structure**					**R**				**Tbr**				**Tcr**			**Ldon**		**Pfc**	**L6**
**33**	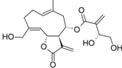					-				5.885 (7.8)				4.775 (0.6)			4.818 (0.7)		5.199 (1.6)	4.991
**34**	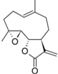					-				6.411 (18.7)				4.972 (0.7)			5.449 (0.3)		4.925 (0.6)	5.140
**35**	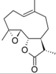					-				4.310 (2.7)				n.a.			4.449 (0.4)		n.a.	3.877
**36**	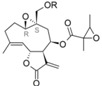					H				4.845 (1.0)				4.592 (0.5)			5.564		5.025 (1.4)	4.866
**37**						ac				6.321 (16.2)				4.802 (0.5)			5.285		5.198 (1.2)	5.111
**Cpd**	**Structure**					**R_1_**		**R_2_**		**Tbr**				**Tcr**			**Ldon**		**Pfc**	**L6**
**38**	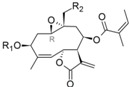					H		H		6.026 (13.8)				4.783 (0.8)			4.740		5.055 (1.5)	4.887
**39**						ac		H		6.095 (12.6)				4.755 (0.6)			4.522		5.093 (1.3)	4.996
**40**						H		Oac		6.156 (9.5)				4.927 (0.6)			4.767		5.178 (1.0)	5.176

**ac =** acetyl; **tig =** tigloyl; **n.a. =** pIC_50_ not available.

### 3.2. Fragment-Based Strategy

The HQSAR technique was chosen as fragment-based drug design strategy [20,21,22,23]. This technique has been successfully employed in drug design studies obtaining good agreement with experimental data of several different compound datasets [24,25,26,27]. The HQSAR technique consists in the decomposition of each molecule in the dataset into a molecular hologram that consists basically of linear, branched, and overlapping fragments which are divided to a fixed-length array (53 to 401 bins). The bin occupancies encode compositional and topological molecular information used as independent (X) variables in QSAR modeling. The hologram length, fragment size and fragment distinction (atoms (A), bonds (B), connections (C), hydrogen atoms (H), chirality (Ch), and H-bond donor/acceptor groups (DA)) are the parameters that affect the hologram generation and consequently the statistical evaluation of constructed HQSAR models. Initially, the several models applying different combinations of fragment distinctions were generated using default fragment size 4–7 atoms over the 13 default series of hologram lengths. Next, the influence of fragment size was further investigated for the best model. All models generated in this study were generated using the Partial Least Squares (PLS) method. Each model was fully cross-validated by the Leave-One-Out (LOO) method.

### 3.3. QSAR Model Validation

After the obtainment of an optimum HQSAR model for each biological activity, we carried out a robustness test and external validation, with a test set of compounds which were not considered for the purpose of QSAR model development [28,29,30]. The robustness test was performed employing the cross-validation (CV) method with pre-determined groups of compounds (from 5 to 25 groups) used to perform the internal capacity of biological activity prediction. All CV validations were carried out in triplicate and the average *q^2^* (coefficient of determination of the predicted *vs.* experimental values during cross validation) and standard deviation for each number of CV groups were also calculated.

Next, the models were submitted to an external validation to estimate the capacity of biological activity prediction for compounds that were not used in HQSAR model construction. Both, residuals of predicted values as well the external validation coefficient (*r^2^_test_ =* coefficient of determination of predicted *vs.* experimental data of the test set) were analyzed in this step. As the pIC_50_ range of the five constructed models is different due to the employed dependent variable (pIC_50_ values for four protozoa and cytotoxicity), the five test sets were selected according to the pIC_50_ distribution of each specific dataset in order to optimally cover the different ranges of biological activity in the dataset.

## 4. Conclusions

All HQSAR models constructed in this study showed good internal consistency and external predictivity. The quality of all models with respect to internal and external predictiveness was evaluated by statistical parameters such as as leave-one-out cross-validation *q^2^* (ranging from 0.637 to 0.775) and quality of test set predictions *r^2^_test_* (ranging from 0.653 to 0.944), respectively. All of the obtained values were above those considered acceptable in literature [31]. All constructed models showed good internal (leave-one-out *q^2^* values ranging from 0.637 to 0.775) and external validation coefficients (*r^2^_test_* values ranging from 0.653 to 0.944). While it was not possible so far to obtain reliable and statistically sound QSAR models for these STLs’ bioactivity against *T. cruzi*, *L. donovani* and *P. falciparum* with classical approaches, this task could now be achieved by using the HQSAR approach.

Apart from their explanatory value, these models can now be used for activity predictions with larger databases of hitherto untested STLs in order to select promising candidates for testing against the parasites under study. It should not remain unmentioned, that recently, Schmidt *et al.* [14] reported on a refined QSAR model for anti-Tbr activity based on an extended data set of almost 70 compounds which could successfully be used to predict very high *in vitro* activity for a group of hitherto untested STLs. By using a similar approach in further studies with the HQSAR models generated in the present work, we expect to find new and promising hits against *T. cruzi*, *L. donovani* and *P. falciparum* within the vast structural diversity of STLs.

## Supplementary Materials

Supplementary materials can be accessed at: http://www.mdpi.com/1420-3049/19/7/10546/s1.
