# Supplementary material for: Hologram QSAR Studies of Antiprotozoal Activities of Sesquiterpene Lactones
Source: Molecules. 2014 Jul 18;19(7):10546–62. doi: 10.3390/molecules190710546 (PMC6271290; doi:10.3390/molecules190710546)
Supplement: Supplementary file 1 [file molecules-19-10546-s001.pdf]

Supplementary Materials

Figure S1. HQSAR maps of compounds 19, 24, 25 and 28.

**Tbr HQSAR model**	**L6 HQSAR model**
**Cpd 24 (SI = 52.1)**	
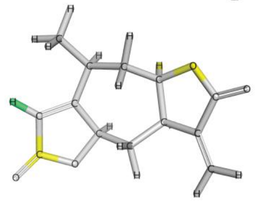	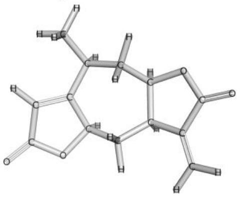
pIC_{50} = 5.086	pIC_{50} = 3.369
**Cpd 26 (SI = 0.1)**	
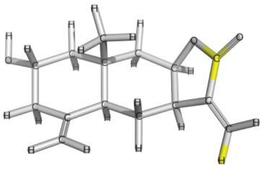	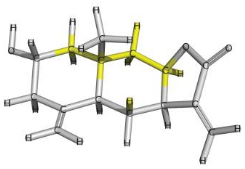
pIC_{50} = 5.108	pIC_{50} = 6.023
**Cpd 25 (SI = 0.2)**	
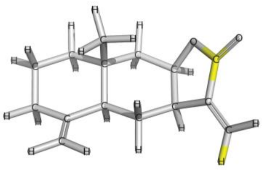	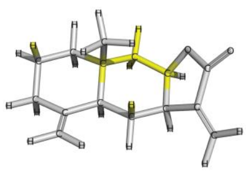
pIC_{50} = 4.627	pIC_{50} = 5.348
**Cpd 28 (SI = 0.5)**	
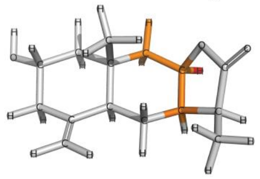	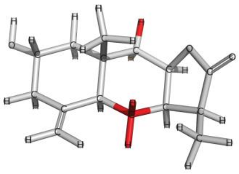
pIC_{50} = 3.467	pIC_{50} = 3.728

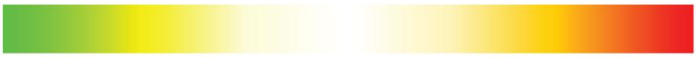

Positive Contribution *Negative Contribution*

Table S1. All constructed HQSAR models with Tbr biological activity.

**Fragment Size = 4 to 7 atoms**						
**F_{dist}**	**q^2**	**SEV**	**r^2**	**SEE**	**HL**	**PC**
A/B	0.459	0.732	0.852	0.383	353	6
**A/B/C**	**0.637**	**0.576**	**0.822**	**0.404**	**53**	**4**
A/B/C/Ch	0.573	0.625	0.833	0.391	307	4
A/B/C/DA	0.543	0.647	0.822	0.404	53	4
A/B/C/H	0.453	0.736	0.893	0.326	151	6
A/B/C/H/Ch	0.549	0.655	0.902	0.306	307	5
A/B/C/H/Ch/DA	0.552	0.653	0.878	0.341	53	5
A/B/C/H/DA	0.528	0.670	0.852	0.376	53	5
A/B/H	0.190	0.845	0.618	0.580	53	3
A/B/H/Ch/DA	0.476	0.720	0.931	0.262	199	6
A/B/H/DA	0.240	0.818	0.649	0.556	83	3
A/C	0.577	0.622	0.835	0.389	71	4
A/C/Ch	0.619	0.591	0.823	0.403	83	4
A/C/Ch/DA	0.601	0.604	0.871	0.343	53	4
A/C/DA	0.531	0.668	0.871	0.351	151	5
A/C/H/Ch	0.491	0.710	0.909	0.300	353	6
**Fragment Distinction = A/B/C**						
**F_{size}**	**q^2**	**SEV**	**r^2**	**SEE**	**HL**	**PC**
1 to 4	0.407	0.736	0.708	0.517	97	4
2 to 5	0.547	0.644	0.761	0.467	83	4
3 to 6	0.546	0.644	0.808	0.419	97	4
5 to 8	0.588	0.614	0.833	0.391	307	4
6 to 9	0.565	0.631	0.817	0.409	53	4
7 to 10	0.519	0.663	0.826	0.398	61	4
8 to 11	0.480	0.690	0.819	0.406	151	4

Table S2. All constructed HQSAR models with Tcr biological activity.

Fragment Size = 4 to 7 atoms						
**F_{dist}**	***q^2***	**SEV**	***r^2***	**SEE**	**HL**	**PC**
A/B	0.695	0.303	0.884	0.187	353	5
A/B/C	0.697	0.309	0.950	0.125	53	6
A/B/C/Ch	0.617	0.332	0.900	0.170	401	4
A/B/C/DA	0.694	0.304	0.922	0.153	71	5
A/B/C/H	0.508	0.394	0.946	0.131	353	6
A/B/C/H/Ch	0.448	0.418	0.942	0.136	353	6
A/B/C/H/Ch/DA	0.449	0.399	0.867	0.196	71	4
A/B/C/H/DA	0.576	0.366	0.924	0.155	71	6
A/B/H	0.371	0.417	0.664	0.305	199	3
A/B/H/Ch/DA	0.370	0.417	0.689	0.293	401	3
A/B/H/DA	0.391	0.410	0.684	0.296	401	3
**A/C**	**0.721**	**0.297**	**0.939**	**0.139**	**71**	**6**
A/C/Ch	0.667	0.317	0.919	0.156	151	5
A/C/Ch/DA	0.643	0.321	0.910	0.161	401	4
A/C/DA	0.665	0.318	0.904	0.170	71	5
A/C/H/Ch	0.391	0.439	0.933	0.145	151	6
**Fragment Distinction = A/C**						
**F_{size}**	***q^2***	**SEV**	***r^2***	**SEE**	**HL**	**PC**
1 to 4	0.330	0.440	0.640	0.322	53	4
2 to 5	0.518	0.381	0.825	0.230	53	5
3 to 6	0.637	0.339	0.934	0.145	199	6
5 to 8	0.741	0.280	0.948	0.126	151	5
6 to 9	0.729	0.286	0.953	0.120	151	5
**7 to 10**	**0.748**	**0.282**	**0.965**	**0.106**	**151**	**6**
8 to 11	0.689	0.299	0.923	0.149	353	4

Table S3. All constructed HQSAR models with Ldon biological activity.

Fragment Size = 4 to 7 atoms						
**F_{dist}**	**q^2**	**SEV**	**r^2**	**SEE**	**HL**	**PC**
A/B	0.731	0.289	0.920	0.158	71	4
A/B/C	0.657	0.336	0.932	0.150	199	5
A/B/C/Ch	0.675	0.335	0.947	0.135	97	6
A/B/C/DA	0.695	0.325	0.961	0.116	59	6
A/B/C/H	0.486	0.390	0.749	0.273	61	3
A/B/C/H/Ch	0.612	0.366	0.963	0.113	59	6
A/B/C/H/Ch/DA	0.642	0.352	0.973	0.096	71	6
A/B/C/H/DA	0.518	0.388	0.821	0.236	61	4
A/B/H	0.545	0.376	0.856	0.212	59	4
A/B/H/Ch/DA	0.524	0.395	0.907	0.174	151	5
A/B/H/DA	0.616	0.365	0.962	0.115	257	6
A/C	0.727	0.292	0.892	0.183	61	4
A/C/Ch	0.707	0.319	0.970	0.103	199	6
**A/C/Ch/DA**	**0.775**	**0.279**	**0.972**	**0.098**	**83**	**6**
A/C/DA	0.768	0.283	0.959	0.119	61	6
A/C/H/Ch	0.477	0.394	0.822	0.230	353	3
**Fragment Distinction = A/C/Ch/DA**						
**F_{size}**	**q^2**	**SEV**	**r^2**	**SEE**	**HL**	**PC**
1 to 4	0.542	0.398	0.881	0.203	151	6
2 to 5	0.618	0.364	0.904	0.182	83	6
3 to 6	0.706	0.319	0.966	0.108	151	6
5 to 8	0.770	0.282	0.982	0.080	199	6
6 to 9	0.747	0.296	0.976	0.091	199	6
7 to 10	0.733	0.296	0.968	0.103	307	5
8 to 11	0.716	0.305	0.965	0.106	257	5

Table S4. All constructed HQSAR models with Pfc biological activity.

**Fragment Size = 4 to 7 atoms**						
**F_{dist}**	**q^2**	**SEV**	**r^2**	**SEE**	**HL**	**PC**
A/B	0.661	0.256	0.859	0.165	199	4
A/B/C	0.684	0.262	0.951	0.104	307	6
A/B/C/Ch	0.637	0.265	0.903	0.137	401	4
A/B/C/DA	0.668	0.253	0.888	0.147	151	4
A/B/C/H	0.545	0.296	0.833	0.180	353	4
A/B/C/H/Ch	0.513	0.307	0.824	0.184	53	4
A/B/C/H/Ch/DA	0.547	0.304	0.899	0.144	53	5
A/B/C/H/DA	0.591	0.281	0.844	0.174	307	4
A/B/H	0.519	0.305	0.822	0.185	257	4
A/B/H/Ch/DA	0.527	0.302	0.820	0.186	199	4
A/B/H/DA	0.528	0.302	0.829	0.181	401	4
**A/C**	**0.703**	**0.254**	**0.950**	**0.104**	**83**	**6**
A/C/Ch	0.682	0.263	0.948	0.106	61	6
A/C/Ch/DA	0.668	0.260	0.947	0.104	199	5
A/C/DA	0.676	0.265	0.954	0.100	151	6
A/C/H/Ch	0.490	0.305	0.763	0.208	199	3
**Fragment Distinction = A/C**						
**F_{size}**	**q^2**	**SEV**	**r^2**	**SEE**	**HL**	**PC**
1 to 4	0.458	0.333	0.809	0.197	59	5
2 to 5	0.615	0.280	0.875	0.160	61	5
3 to 6	0.730	0.242	0.944	0.110	71	6
5 to 8	0.706	0.253	0.961	0.092	307	6
6 to 9	0.683	0.262	0.965	0.087	353	6
**7 to 10**	**0.736**	**0.232**	**0.960**	**0.090**	**97**	**5**
8 to 11	0.732	0.241	0.982	0.063	353	6

Table S5. All constructed HQSAR models with L6 biological activity.

**Fragment Size = 4 to 7 atoms**						
**F_{dist}**	***q^2***	**SEV**	***r^2***	**SEE**	**HL**	**PC**
A/B	0.603	0.371	0.869	0.213	97	6
A/B/C	0.629	0.351	0.862	0.215	353	5
A/B/C/Ch	0.646	0.351	0.907	0.18	61	6
A/B/C/DA	0.621	0.355	0.852	0.222	97	5
A/B/C/H	0.633	0.357	0.912	0.175	353	6
A/B/C/H/Ch	0.524	0.407	0.91	0.177	353	6
A/B/C/H/Ch/DA	0.543	0.398	0.924	0.162	53	6
A/B/C/H/DA	0.609	0.368	0.917	0.169	401	6
A/B/H	0.518	0.409	0.873	0.21	353	6
A/B/H/Ch/DA	0.444	0.439	0.897	0.189	401	6
A/B/H/DA	0.565	0.389	0.896	0.19	353	6
A/C	0.636	0.348	0.852	0.222	257	5
A/C/Ch	0.629	0.351	0.88	0.2	199	5
**A/C/Ch/DA**	**0.647**	**0.343**	**0.893**	**0.189**	**61**	**5**
A/C/DA	0.582	0.365	0.844	0.223	353	4
A/C/H/Ch	0.544	0.398	0.914	0.173	353	6
**Fragment Distinction = A/C/Ch/DA**						
**F_{size}**	***q^2***	**SEV**	***r^2***	**SEE**	**HL**	**PC**
1 to 4	0.204	0.504	0.713	0.303	61	4
2 to 5	0.465	0.422	0.856	0.219	71	5
3 to 6	0.568	0.371	0.843	0.224	307	4
**5 to 8**	**0.673**	**0.337**	**0.952**	**0.129**	**71**	**6**
6 to 9	0.619	0.363	0.966	0.109	59	6
7 to 10	0.549	0.379	0.889	0.188	151	4
8 to 11	0.583	0.373	0.948	0.131	151	5

Table S6. Experimental and predicted pIC_{50} values of all constructed HQSAR models and its residues of prediction values.

	**Tbr HQSAR Model**			**Tcr HQSAR Model**			**Ldon HQSAR Model**			**Pfc HQSAR Model**			**L6 HQSAR Model**		
**cpd**	**exp**	**pred**	**res**	**exp**	**pred**	**res**	**exp**	**pred**	**res**	**exp**	**pred**	**res**	**Exp**	**pred**	**res**
**1**	7.28	6.68	−0.60	6.16 [ts]	5.66	−0.50	n.a.	-	-	n.a.	-	-	6.00 [ts]	5.80	−0.20
**2**	7.20	7.11	−0.09	6.27	5.82	−0.45	6.35	5.99	−0.36	6.48	5.99	−0.50	6.09	5.97	−0.12
**3**	6.98 [ts]	7.07	0.09	5.80	6.33	0.53	6.08	6.25	0.17	6.15 [ts]	6.31	0.16	5.99	6.01	0.02
**4**	6.94	7.17	0.23	5.61 [ts]	6.03	0.43	6.06	6.22	0.16	6.09	6.44	0.36	5.89	6.03	0.14
**5**	6.16	5.84	−0.32	4.67	4.95	0.28	5.41	5.56	0.15	5.52	5.66	0.15	5.05	4.89	−0.16
**6**	5.85	6.32	0.47	5.16	5.31	0.15	n.a.	-	-	n.a.	-	-	5.51 [ts]	5.42	−0.09
**7**	6.04	6.08	0.04	5.45	5.16	−0.29	5.83 [ts]	6.01	0.18	5.80 [ts]	5.98	0.18	5.34	5.29	−0.04
**8**	6.50	6.93	0.43	5.73	5.51	−0.22	n.a.	-	-	n.a.	-	-	5.61	5.64	0.03
**9**	5.03	5.30	0.27	4.34	4.32	−0.02	n.a.	-	-	n.a.	-	-	4.91	4.88	−0.03
**10**	5.17	4.96	−0.21	4.69	4.53	−0.16	4.91 [ts]	4.90	−0.01	5.19	5.00	−0.19	5.36	4.73	−0.63
**11**	4.74	4.67	−0.07	4.28	4.37	0.10	4.69	4.67	−0.02	4.92	4.95	0.03	4.50	4.85	0.36
**12**	4.96	5.48	0.52	4.31	4.63	0.32	4.75 [ts]	4.84	0.10	4.97	4.72	−0.25	4.78	5.00	0.23
**13**	4.72 [ts]	5.30	0.58	4.22 [ts]	4.53	0.31	4.84	4.78	−0.06	4.56	5.07	0.50	4.50 [ts]	4.73	0.23
**14**	5.93	4.92	−1.01	4.83	4.80	−0.03	5.37	5.01	−0.36	5.05 [ts]	5.36	0.31	5.07	5.11	0.04
**15**	5.40 [ts]	5.59	0.19	4.79	4.73	−0.05	5.06	5.41	0.35	5.22 [ts]	5.27	0.05	5.14	5.10	−0.04
**16**	4.87	5.16	0.29	4.35	4.47	0.12	4.93	4.91	−0.02	4.97 [ts]	5.40	0.43	4.67	5.27	0.61
**17**	4.21 [a]	-	-	n.a.	-	-	4.62	4.80	0.17	n.a.	-	-	3.75 [ts]	4.49	0.74
**18**	5.53	5.80	0.27	4.58	4.81	0.23	4.75	4.65	−0.10	5.11	5.12	0.01	4.52	4.95	0.43
**19**	6.48 [ts]	5.51	−0.98	4.95 [ts]	4.76	−0.19	6.22 [a]	-	-	5.19	5.22	0.03	4.65	4.99	0.33
**20**	4.80	4.68	−0.12	4.41	4.69	0.28	4.84	4.89	0.06	4.90	5.13	0.24	4.70	4.91	0.21
**21**	6.20	6.32	0.12	4.79	4.59	−0.20	4.66	4.66	0.00	5.30	5.37	0.07	5.03 [ts]	4.98	−0.05
**22**	5.89	5.86	−0.03	4.02	4.44	0.42	4.57	4.80	0.23	5.19	5.26	0.08	4.88	5.07	0.20
**23**	3.79	4.45	0.66	n.a.	-	-	4.81 [ts]	5.08	0.27	n.a.	-	-	n.a.	-	-
**24**	5.09	6.03	0.94	n.a.	-	-	4.57	4.84	0.27	n.a.	-	-	3.37 [a]	-	-
**25**	4.63 [ts]	5.11	0.49	4.65 [ts]	4.64	−0.01	n.a.	-	-	n.a.	-	-	5.35	4.86	−0.49
**26**	5.11	5.12	0.01	4.61	4.60	−0.01	n.a.	-	-	n.a.	-	-	6.02 [a]	-	-
**27**	4.97	5.38	0.41	4.61	4.67	0.06	4.93	5.32	0.39	5.04	5.12	0.09	5.05	4.99	−0.06
**28**	3.47	4.33	0.87	n.a.	-	-	n.a.	-	-	n.a.	-	-	3.73	4.44	0.72
**29**	5.58	5.37	−0.21	5.08	4.67	−0.41	5.52	5.16	−0.36	5.19	5.21	0.02	5.22 [ts]	4.86	−0.36
**30**	5.80 [ts]	5.55	−0.25	4.76	5.14	0.38	5.06	5.31	0.25	4.97	5.05	0.08	4.99	4.84	−0.16
**31**	4.75	5.32	0.57	4.54 [ts]	4.57	0.03	5.22	4.98	−0.25	4.92	4.85	−0.07	4.48	4.67	0.19
**32**	5.96	5.02	−0.94	4.58	4.77	0.19	5.13	5.07	−0.07	4.87 [ts]	4.82	−0.05	4.59	4.50	−0.09
**33**	5.89	5.77	−0.12	4.77	4.80	0.03	4.82	4.83	0.01	5.20	5.12	−0.08	4.99	4.77	−0.22
**34**	6.41	5.60	−0.81	4.97	4.79	−0.18	5.45 [a]	-	-	4.92	5.12	0.20	5.14	4.93	−0.21
**35**	4.31 [ts]	5.16	0.85	n.a.	-	-	4.45	4.68	0.23	n.a.	-	-	3.88	4.44	0.56
**36**	4.85	5.77	0.92	4.59	4.72	0.12	5.56	5.06	−0.51	5.02	5.18	0.16	4.87 [ts]	5.12	0.26
**37**	6.32	5.37	−0.95	4.80	4.67	−0.13	5.28 [ts]	5.51	0.23	5.20	5.16	−0.04	5.11	5.08	−0.03
**38**	6.03	5.70	−0.33	4.78 [ts]	4.76	−0.02	4.74	4.76	0.02	5.05	5.05	0.00	4.89	4.90	0.02
**39**	6.09	5.81	−0.29	4.76	4.88	0.13	4.52 [ts]	4.80	0.28	5.09	5.11	0.02	5.00	5.02	0.02
**40**	6.16 [ts]	5.85	−0.30	4.93	4.77	−0.16	4.77	5.01	0.24	5.18	5.09	−0.09	5.18	5.17	0.00

exp - experimental pIC_{50}; **pred** - predicted pIC_{50}; **res** - residue of prediction (pred - exp); **n.a.** - pIC_{50} not available; **a** - compound considered outlier; **[ts]** - test set compound.

Table S7. Correlation between antiprotozoal and citotoxic activities (correlation coefficients of pIC_{50} data) presented by Schmidt *et al.* [1].

	**Tbr**	**Tcr**	**Ldon**	**Pfc**	**L6**
**Tbr**	1.000				
**Tcr**	0.851	1.000			
**Ldon**	0.704	0.823	1.000		
**Pfc**	0.808	0.912	0.753	1.000	
**L6**	0.698	0.857	0.697	0.905	1.000

References

1. Schmidt, T.; Nour, A.; Khalid, S.; Kaiser, M.; Brun, R. Quantitative structure-antiprotozoal activity relationships of sesquiterpene lactones. *Molecules* **2009**, *14*, 2062–2076.
